# Yeast *RAD2*, a homolog of human *XPG*, plays a key role in the regulation of the cell cycle and actin dynamics

**DOI:** 10.1242/bio.20136403

**Published:** 2013-12-04

**Authors:** Mi-Sun Kang, Sung-Lim Yu, Ho-Yeol Kim, Choco Michael Gorospe, Byung Hyune Choi, Sung Haeng Lee, Sung-Keun Lee

**Affiliations:** 1 Department of Pharmacology, College of Medicine, Inha University, Incheon, Korea 400-712; 2 Inha Research Institute for Medical Sciences, College of Medicine, Inha University, Incheon, Korea 400-712; 3 Department of Molecular Biomedicine, College of Medicine, Inha University, Incheon, Korea 400-712; 4 Division of Biomedical and Bioengineering Sciences, College of Medicine, Inha University, Incheon, Korea 400-712; 5 Department of Cellular and Molecular Medicine, Chosun University School of Medicine, Gwangju, Korea 501-759

**Keywords:** *RAD2*, Cell cycle, Actin dynamics

## Abstract

Mutations in the human *XPG* gene cause Cockayne syndrome (CS) and xeroderma pigmentosum (XP). Transcription defects have been suggested as the fundamental cause of CS; however, defining CS as a transcription syndrome is inconclusive. In particular, the function of XPG in transcription has not been clearly demonstrated. Here, we provide evidence for the involvement of *RAD2*, the *Saccharomyces cerevisiae* counterpart of *XPG*, in cell cycle regulation and efficient actin assembly following ultraviolet irradiation. *RAD2* C-terminal deletion, which resembles the *XPG* mutation found in XPG/CS cells, caused cell growth arrest, the cell cycle stalling, a defective α-factor response, shortened lifespan, cell polarity defect, and misregulated actin-dynamics after DNA damage. Overexpression of the C-terminal 65 amino acids of Rad2p was sufficient to induce hyper-cell polarization. In addition, *RAD2* genetically interacts with *TPM1* during cell polarization. These results provide insights into the role of *RAD2* in post-UV irradiation cell cycle regulation and actin assembly, which may be an underlying cause of XPG/CS.

## Introduction

DNA is subject to constant damage by various factors including ultraviolet (UV) light. Bulky nucleotide adducts in UV-damaged DNA are mainly repaired by the nucleotide excision repair (NER) system. UV damage in the transcribed strand is removed faster than damage in the non-transcribed strand through a sub-pathway of NER called transcription-coupled NER (TC-NER). Repair of non-transcribed DNA region is called global genomic NER (GG-NER) (Friedberg et al., 2006). The difference between these two NER sub-pathways is the initial damage recognition step. DNA damage is recognized by RNA polymerase during transcription in the TC-NER whereas DNA damage recognition proteins detect damages in GG-NER. Regardless of the different repair rates, both pathways require common components to complete the repair process. Once damaged DNA lesions are recognized, an essential RNA polymerase transcription factor II (TFIIH) that contains two DNA helicases, XPB and XPD, unwinds the DNA helix. Then, the lesion-containing nucleotides are removed *via* incision on both sides of the lesions by two NER-specific endonucleases, XPF-ERCC1 and XPG, and the gaps are resynthesized by replicative DNA synthesis (Hoeijmakers, 2001; Nouspikel, 2009).

Cells with a compromised DNA repair system are confronted with irreversible mutations that can result in deleterious events such as cell death, aging, and cancer (Bartek and Lukas, 2007; Rouse and Jackson, 2002). Mutations in 8 NER-related genes cause xeroderma pigmentosum (XP), a genetic disorder characterized by extreme sensitivity to sunlight, abnormal pigmentation, and a striking increase in skin cancer incidence (Kraemer and DiGiovanna, 2003 (http://www.ncbi.nlm.nih.gov/books/NBK1397)). NER defects cause another human hereditary disorder Cockayne syndrome (CS) that is characterized by growth and mental retardation, impaired neurological development and premature aging without increased cancer predisposition. Although both TC-NER and GG-NER require NER components, mutations only in 3 NER genes, XPB, XPD and XPG, cause CS (Hanawalt, 2000). *XPB* and *XPD* encode components of TFIIH that function in NER and transcription (Egly and Coin, 2011). Mutations in two other genes, *CSA* and *CSB*, also cause CS. CSB is a DNA-dependent ATPase that participates in transcription by stimulating transcription elongation (Selby and Sancar, 1997) and transcription initiation of some genes after UV-damage (Proietti-De-Santis et al., 2006). CSA is required for recruitment of transcription elongation factor S in cooperation with CSB (Fousteri et al., 2006). Compared to the clear transcription functions of these four CS-related genes, the role of XPG was unknown before a report on *RAD2*, a yeast homolog of human *XPG*, showed that it functions in transcription elongation. Both XPG and Rad2p are structure-specific endonucleases that cleave 3′ to the DNA lesion (Habraken et al., 1995; O'Donovan et al., 1994), and possess highly conserved N and I domain in the N terminus and in the internal region, respectively. An additional function of Rad2p was determined in a study using a C-terminal truncation mutant of *RAD2* (*rad2*C209Δ) that is analogous to the C-terminal truncation in the XPG of XPG/CS patient (XPCS1RO). The *rad2*C209Δ mutation impaired transcription elongation in the absence of extrinsic DNA damage. The highly conserved structure of human XPG and Rad2p suggested a similar function for XPG in transcription elongation (Lee et al., 2002a). Accordingly, CS is considered a disorder due to defects in transcription (Egly and Coin, 2011; Schärer, 2008a).

Nevertheless, it is still unconvincing to define CS as a transcription syndrome for several reasons. First, some XPD C-terminal mutations alter transcription but are not correlated with XP/CS phenotypes (Dubaele et al., 2003; Schärer, 2008a). Second, although the roles of XPG in transcription have been suggested by a yeast study (Lee et al., 2002a), they have not been clearly demonstrated in higher eukaryotes. In addition, the function of Rad2p in transcription elongation was demonstrated in the absence of extrinsic DNA damage. Because photosensitivity is a fundamental characteristic of CS (Laugel, 2013) and XPG/CS is abruptly aggravated after UV irradiation (Jaeken et al., 1989; Lindenbaum et al., 2001), the function of Rad2p after UV irradiation needs to be verified. Third, although mutations in two TFIIH subunits, p44 and p52, destabilize TFIIH (Schärer, 2008a), they do not cause any known human disease. However, mutations in XPB and XPD, essential TFIIH components, cause CS. Fourth, XP patients with XPC or XPE mutations are TC-NER deficient, but do not display CS symptoms (Nouspikel, 2009). It is particularly difficult to explain how the common features of CS can arise from transcriptional deficiencies in various genes in different CS patients who might be exposed to a variety of environments and develop CS at different ages (Schärer, 2008a). These observations imply that transcription defects are not sufficient to explain the cause of CS and why CS symptoms are not exclusively associated with NER. These observations also suggest that XPG/Rad2p has a yet unidentified function.

While mutations in the I-domain of XPG cause XP, C-terminal deletion mutations cause XPG/CS (Clarkson, 2003). The C-terminal region of XPG has been shown to participate in multiple cellular mechanisms including stable XPG-TFIIH complex formation (Ito et al., 2007), cellular localization (Park et al., 1996), and interaction with multiple partners (Trego et al., 2011). XPG interacts with TFIIH components such as XPB, XPD, p66, and p44 through amino acids 747–928 (Iyer et al., 1996). However, the smallest XPG C-terminal deletion found in XPG/CS cell is 258 amino acids (926–1,186) found in the XPCS1RO patient (Nouspikel et al., 1997) in which XPG and TFIIH are dissociated (Ito et al., 2007). In summary, there are discrepancies between the known TFIIH binding sites of XPG and the mutations in XPG/CS cells that cause XPG-TFIIH dissociation. Therefore, more investigation is needed to clarify the reason for such disagreement. A study of mouse XPG deletion mutants indicated that the absence of the proliferating cell nuclear antigen-binding domain (PCNA-BD) is not responsible for CS phenotypes (Shiomi et al., 2004). Therefore, if the XPG/Rad2p C-terminal region has a yet unknown function, the C-terminal amino acids, between 926 and N-terminal to the PCNA-BD of XPG, are likely to be responsible for it (supplementary material Fig. S1B).

Some data indicate that CS is caused by a factor related to cellular functions other than transcription. Because TFIIH is a multifunctional complex involved in basal transcription, cell cycle regulation, and NER, CS-inducing mutations in XPB and XPD have the potential to induce defects in other cellular processes. Indeed, recent studies have shown that XPB and XPD function in cell cycle regulation (Ito et al., 2010; Fuss and Tainer, 2011). XPG has also been suggested to have unknown S phase-specific functions in concert with other unknown protein partners (Trego et al., 2011). Moreover, the cell growth arrest and mitotic catastrophe induced by overexpression of yeast *RAD2* alone suggest a role for *RAD2* in cell cycle regulation (Kang et al., 2010).

Cells exposed to DNA damaging agents express many genes to arrest the cell cycle progression and deal with DNA damage (Enserink et al., 2006; Weinert, 1998). Cell cycle arrest is commonly accompanied by cell polarity arrest, which is required for morphogenesis and development in higher eukaryotes (Formigli et al., 2007). Therefore, unattainable re-initiation of the cell cycle from damage-induced cell cycle arrest is not possible growth and viability. Interestingly, CS symptoms such as growth retardation and premature aging, can arise when cell cycle progression is hampered. Taken together, mechanisms other than transcription, especially cell cycle regulation, might be responsible for CS.

In an effort to settle the controversial role of the XPG/*RAD2* C-terminal region in the pathogenesis of CS, we attempted to investigate the unknown functions of yeast *RAD2*. We analyzed multiple *rad2* C-terminal deletion (*rad2*CΔ) mutants to define the sequences responsible for the CS-like phenotypes and examine the roles of those sequences in various cellular mechanisms. In the absence of UV irradiation, *rad2*CΔ mutations did not cause any notable changes. However, UV irradiation of these mutants arrested cell growth and the cell cycle. Further analysis revealed that the Rad2p C-terminal region is not only essential for cell cycle regulation but also modulation of cell polarization through actin dynamics after UV irradiation. We discuss the relevance of our results in XPG/CS pathogenesis through cell cycle defects and improper control of actin dynamics after DNA damage.

## Results

### *rad2*CΔ mutations cause cell growth arrest after UV irradiation

**Fig. 1.**
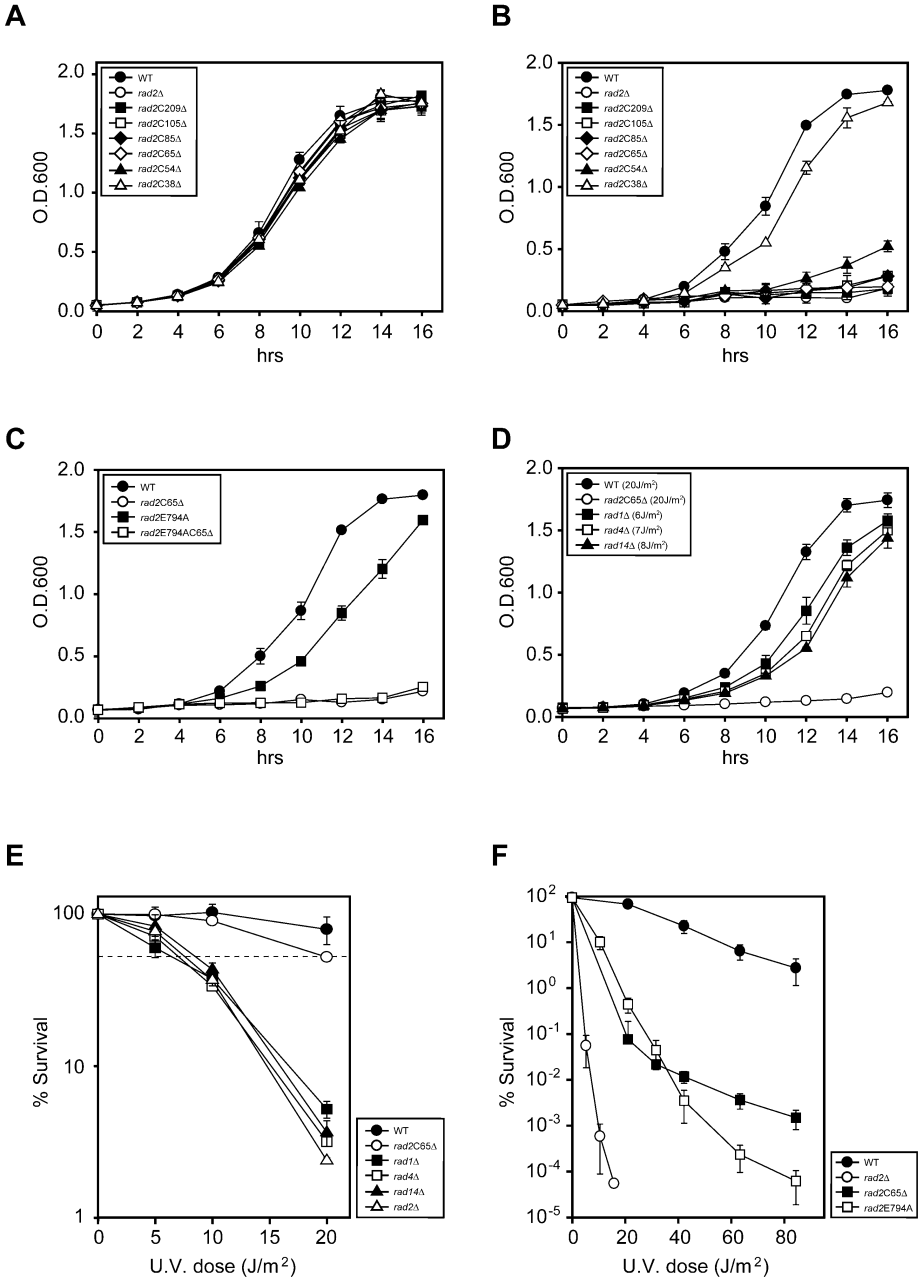
Growth of WT and mutant strains. (A) In the absence of UV irradiation, cell growth of all *rad2* mutants was comparable to that of WT. (B) After 20 J/m^2^ of UV irradiation, the cell growth of all *rad2*CΔ mutants, except *rad2*C38Δ, was impaired. (C) After of UV irradiation, growth of *rad2*E794A was initially somewhat impaired; however, at 16 hours, its growth was comparable to that of WT. *rad2*E794AC65Δ refers the mutant with both the *rad2*E794A and *rad2*C65Δ mutation. (D) Cell growth of *rad1*Δ, *rad4*Δ, and *rad14*Δ mutants irradiated with equivalent toxic UV doses of 6, 7, and 8 J/m^2^ of UV, respectively, was elevated to levels significantly higher than that of *rad2*C65Δ mutant irradiated with 20 J/m^2^. (E) UV sensitivity of *rad1*Δ, *rad4*Δ, and *rad14*Δ mutants. The dotted line indicates 70% viability. (F) The survival rate of the *rad2*E794A mutant showed a linear decrease with increasing UV radiation. The survival rate of the *rad2*C65Δ mutant clearly decreased in response to 20 J/m^2^ UV radiation, but the dose-dependence of this decrease was significantly reduced at UV doses higher than 20 J/m^2^. Each curve represents an average of 3 or more experiments.

The abrupt onset of CS symptoms after UV exposure in some CS patients who were normal at birth (Jaeken et al., 1989; Lindenbaum et al., 2001) suggests that CS is aggravated by UV exposure. To examine the effects of UV irradiation on the development of CS phenotypes, we first compared cell growth before and after UV irradiation in 7 different *rad2* deletion mutants, including those that mimic XPG mutations found in XPG and XPG/CS cells (supplementary material Fig. S1A; Table S1), to wild type (WT). Rad2p and human XPG are highly conserved and C-terminal truncation of XPG causes CS; therefore we expected to observe cellular CS effects, including cell cycle arrest and/or cell growth arrest, in yeast *rad2*CΔ mutants, especially after UV irradiation. All mutants grew comparably well to the WT in the absence of UV irradiation (Fig. 1A), whereas the growth of most *rad2*CΔ and *rad2*Δ mutants was drastically delayed following UV irradiation, (Fig. 1B). Conversely, deletion of the last 38 amino acids (*rad2*C38Δ) did not affect cell growth, even after UV irradiation, indicating that the last 38 amino acids do not contribute to the cell growth arrest of *rad2*CΔ mutants after UV irradiation.

Patients with XPG I domain mutations that abolish endonuclease activity suffer from XP, but do not develop CS (Schärer, 2008b). The *rad2*E794A mutant (*rad2*E794A), which mimics the XPG I domain mutation, grew normally in the absence of UV irradiation (supplementary material Fig. S2). After UV irradiation, the *rad2*E794A mutant initially grew slowly, but it reached levels similar to those of the WT after 16 hours (Fig. 1C). The 54-amino acid deletion mutant of Rad2p had a greatly decreased, but somewhat better growth rate than the mutant with a 65-amino acid deletion in the C-terminus (*rad2*C65Δ); the *rad2*C65Δ mutant still suffered from severe growth inhibition after 16 hours (Fig. 1B). These results indicate that the loss of endonuclease activity of Rad2p is not responsible for UV-induced growth retardation and that the C-terminal ∼38–65 amino acids of Rad2p are crucial to growth regulation after UV irradiation.

We also examined the growth rate of yeast with mutations in other NER genes (*RAD1*, *RAD4*, and *RAD14*, which are the yeast homologs of *XPF*, *XPC*, and *XPA*, respectively) that cause XP but not CS (Kraemer et al., 1987; Kraemer and DiGiovanna, 2003 (http://www.ncbi.nlm.nih.gov/books/NBK1397)). The *rad1*Δ, *rad4*Δ, and *rad14*Δ mutants grew normally in the absence of UV irradiation (supplementary material Fig. S3A). However, after 20 J/m^2^ of UV irradiation, the growth rate of these mutants was almost identical to that of the *rad2*C65Δ mutant (supplementary material Fig. S3B). Because deletions in these 3 genes cause extreme UV sensitivity, we determined the dose for each mutant that induced a toxicity equivalent to 20 J/m^2^ of UV radiation in the *rad2*C65Δ mutant (Fig. 1E). When *rad1*Δ, *rad4*Δ, and *rad14*Δ mutants were irradiated with equivalent toxic UV doses of 6, 7, and 8 J/m^2^ of UV radiation, their growth rates were elevated to levels significantly higher than that of the *rad2*C65Δ mutant irradiated with 20 J/m^2^ (Fig. 1D). These findings indicate that the cell growth retardation of the *rad2*C65Δ mutant after UV irradiation is a unique characteristic of *RAD2*.

The *rad2*Δ, *rad2*E794A, and *rad2*C65Δ mutants were all very sensitive to UV radiation, but the dose-dependent patterns of sensitivity differed greatly. The *rad2*Δ mutant was highly sensitive to low dose UV radiation, and the survival of *rad2*E794A mutant decreased linearly with increasing UV radiation doses. In contrast, survival of the *rad2*C65Δ mutant clearly decreased in response to 20 J/m^2^ UV radiation; however, the dose-dependence of this decrease was significantly reduced at UV doses higher than 20 J/m^2^ (Fig. 1F) (Lee et al., 2002a). These findings indicate that the Rad2p C-terminal region has an unknown function in growth regulation following UV irradiation, and that the UV sensitivity and UV-induced cell growth retardation of the *rad2*C65Δ mutant are not simply the result of an NER defect.

### Cell growth retardation of *rad2*CΔ mutants after UV irradiation is linked to cell cycle arrest

**Fig. 2.**
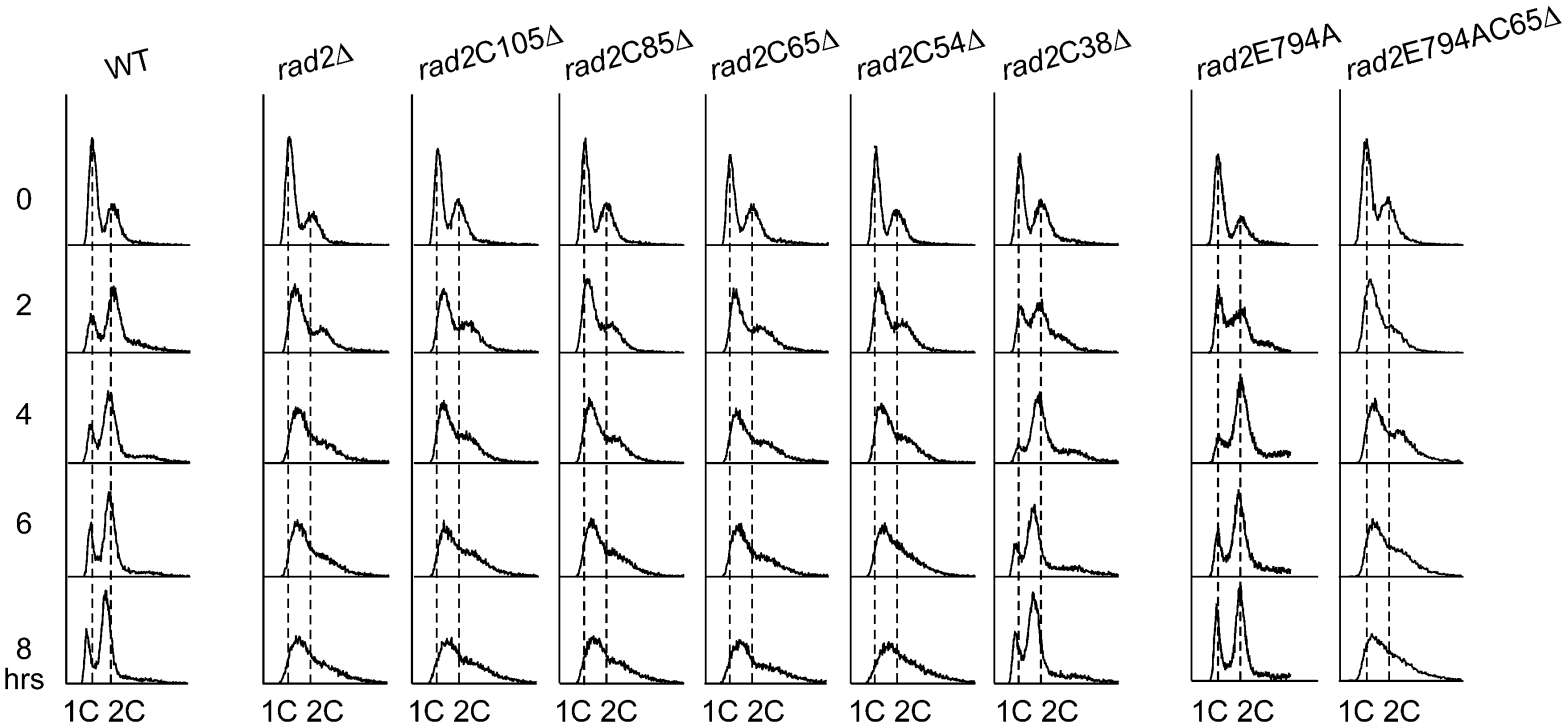
DNA content analysis of WT and *rad2* mutants after UV damage. Strains with a C-terminal of Rad2p, except 38 amino acids deletion, had severely retarded cell cycle progression at G1/S phase in the presence of 20 J/m^2^ UV irradiation, similar to *rad2*Δ mutant. The cells were synchronized by grown to stationary phase before UV irradiation.

Overexpression of *RAD2* induces mitotic catastrophe, suggesting a role in cell cycle regulation (Kang et al., 2010). Therefore, *rad2*CΔ could cause cell cycle arrest and subsequent growth retardation after UV exposure. To verify this hypothesis, *rad2*CΔ, *rad2*E794A, and *rad2*E794AC65Δ mutants were subjected to cell cycle analysis. In the absence of UV irradiation, the cell cycle patterns of all mutants were identical to that of the WT (supplementary material Fig. S4). Following 20 J/m^2^ UV irradiation, the cell cycle of the *rad2*CΔ mutants, other than *rad2*C38Δ, was arrested at G1/S (Fig. 2), suggesting that the cell growth inhibition of the *rad2*CΔ mutants in Fig. 1 was a result of cell cycle arrest. Cell cycle progression of the *rad2*E794A mutant appeared to be temporarily stalled at S phase 2 h after UV irradiation, but then was released shortly thereafter to progress to G2 phase at 4 h. However, the cell cycle of the *rad2*E794AC65Δ mutant was arrested in a fashion similar to that of the *rad2*C65Δ mutant. These results suggest that the C-terminal region of Rad2p is required for release from UV-induced cell cycle arrest.

### The *rad2*CΔ mutant produces large unbudded cells and disorganized actin patches after UV irradiation

**Fig. 3.**
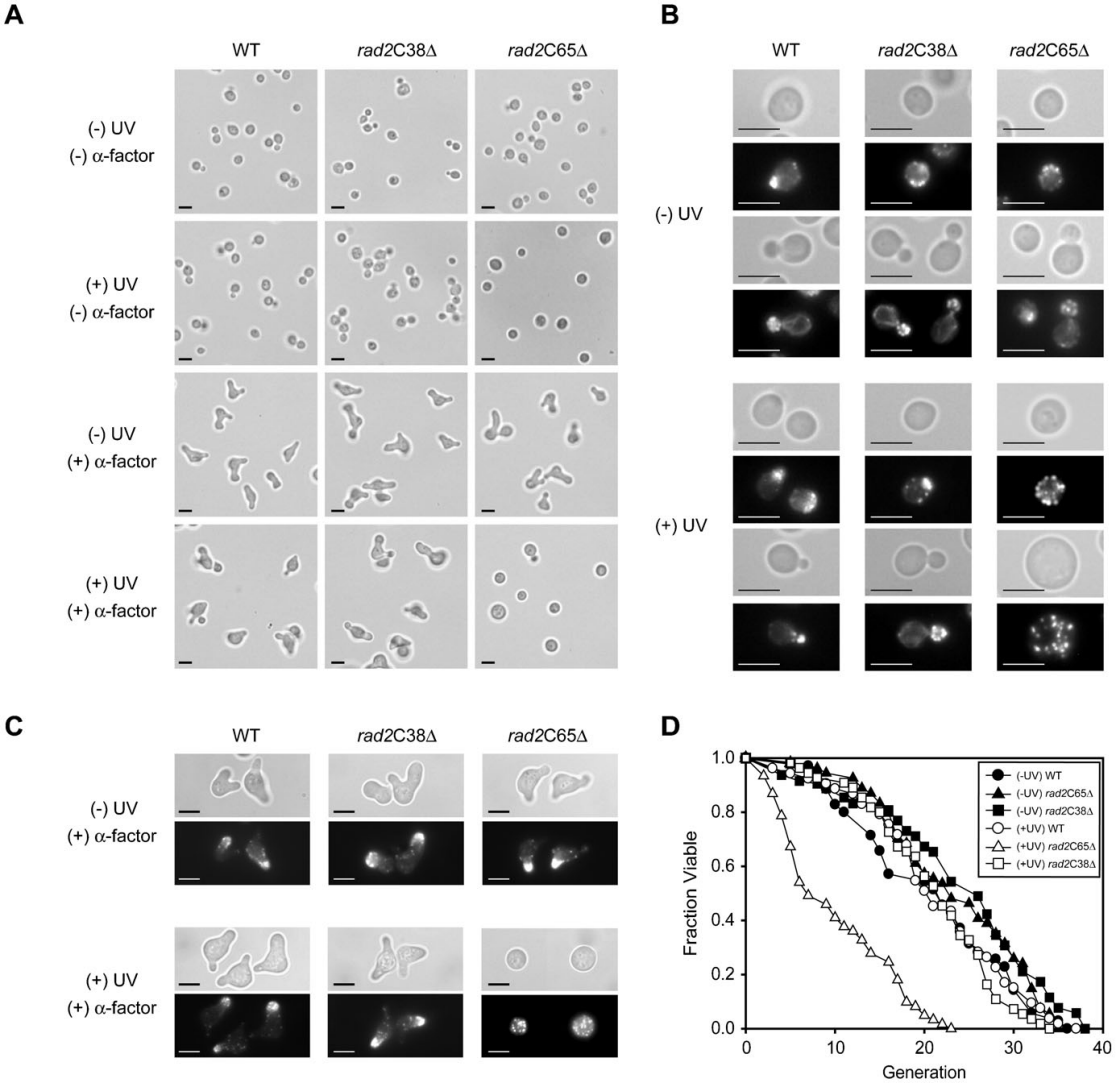
Morphological alterations in the *rad2*C65Δ mutant. (A) Morphogenesis of WT and 2 *rad2*CΔ mutants, *rad2*C38Δ and *rad2*C65Δ, in response to α-factor before and after UV irradiation. Following 20 J/m^2^ UV exposure, the *rad2*C65Δ mutant ceased bud formation and became larger in size than WT. UV irradiation eliminated the α-factor response of *rad2*C65Δ mutant. Bars, 5 µm. (B,C) Rhodamine-phalloidin staining of WT and 2 *rad2*C deletion mutants, *rad2*C38Δ and *rad2*C65Δ. After UV irradiation, the actin in the *rad2*C65Δ mutant was randomly distributed with or without α-factor treatment. Bars, 5 µm. (D) UV irradiation (4 J/m^2^) drastically decreased the replicative lifespan of the *rad2*C65Δ mutant, whereas it did not affect the replicative lifespan of WT and the *rad2*C38Δ mutant.

Yeast cells arrested at a specific point in the cell cycle have characteristic morphologies (Pruyne and Bretscher, 2000a); therefore, the morphological changes in the WT, *rad2*C38Δ, and *rad2*C65Δ mutants were examined. Because the *rad2*C65Δ *rad2*C85Δ, *rad2*C105Δ, and *rad2*C209Δ mutants had identical phenotypes in the cell growth and cell cycle analysis, only 2 *rad2*CΔ mutants, *rad2*C65Δ and *rad2*C38Δ, were used for the rest of the experiments in this study. In the absence of UV exposure, the cell size and cell morphology of the 2 mutants were similar to WT (Fig. 3A,B). However, after UV irradiation, the *rad2*C65Δ mutant had a significantly increased number of cells with a single unbudded morphology characteristic of G1 cells, whereas the WT and *rad2*C38Δ mutant had no significant change in their size and morphology (Fig. 3A,B). After UV irradiation, the number of single enlarged unbudded cells also increased and comprised approximately 22% of the *rad2*C65Δ mutant cells.

The single enlarged unbudded cell shape of the *rad2*C65Δ mutant is similar to the morphology of cells deficient in bud formation due to a defect in Cdc42p, which is essential for yeast actin cytoskeleton polarization (Pruyne and Bretscher, 2000a). Therefore, these findings suggest the involvement of *RAD2* in the regulation of actin cytoskeleton polarity. To examine this possibility, the actin cytoskeleton localization was observed in WT, *rad2*C38Δ, and *rad2*C65Δ mutants. In the absence of UV irradiation, actin patches and actin cables were properly distributed according to the cell cycle stages in all 3 strains (Fig. 3B). After UV irradiation, the WT and *rad2*C38Δ mutant showed no significant change in actin polarization. However, in the *rad2*C65Δ mutant, actin cables were undetectable and actin patches were dispersed in a disorganized pattern throughout the entire cytoplasm (Fig. 3B) similar to those observed in the large unbudded *cdc42* mutant cells (Adams et al., 1990). These results strongly suggest that Rad2p plays a role in actin cytoskeleton polarization after UV irradiation.

### The *rad2*CΔ mutation causes sterility and reduces lifespan after UV irradiation

When actin cytoskeleton dynamics are defective, yeast cells are commonly sterile and do not respond to mating factors. Therefore, if the morphological changes observed in the *rad2*C65Δ mutant are connected to actin cytoskeleton polarization after UV exposure, the *rad2*C65Δ mutant should not respond well to mating factors after UV irradiation. We analyzed the response of WT, *rad2*C38Δ, and *rad2*C65Δ mutants to α-factor before and after UV irradiation. All yeast strains used in this study were a-type cells, therefore, α-factor treatment would cause G1 arrest and mating projection (schmoo) formation. In the absence of UV irradiation, the *rad2*C65Δ and *rad2*C38Δ mutants responded well to 5 µM α-factor, and, similar to WT, approximately 90% of cells formed a shmoo (Fig. 3A). UV irradiation alone did not induce shmooing in any of the strains. When α-factor was applied after UV irradiation, approximately 85% of WT and 80% of the *rad2*C38Δ mutant cells formed a shmoo; however, less than 2% of the *rad2*C65Δ mutants responded to α-factor suggesting that UV irradiation sterilized the *rad2*C65Δ mutant (Fig. 3A).

Shmooing in response to α-factor depends on actin dynamics (Pruyne and Bretscher, 2000a; Pruyne and Bretscher, 2000b); therefore, the sterility of the *rad2*C65Δ mutant after UV irradiation might result from a defect in actin dynamics. To test this hypothesis, actin distribution was analyzed after UV irradiation and α-factor treatment. In the absence of UV irradiation, all 3 strains showed good actin polarization after α-factor treatment. However, after treatment with UV irradiation, actin filaments were randomly distributed in the *rad2*C65Δ mutant following incubation with α-factor (Fig. 3C). These results indicate that the UV-induced sterility of the *rad2*C65Δ mutant is indeed the result of defective actin dynamics due to the deletion of 65 amino acids in the Rad2p C-terminal region.

After UV irradiation, the aging of CS patients is accelerated (Lindenbaum et al., 2001). Therefore, the replicative lifespan of the WT, *rad2*C38Δ, and *rad2*C65Δ mutants was examined before and after UV irradiation. In the absence of UV irradiation, all 3 strains had similar replicative lifespans. However, following UV irradiation at 4 J/m^2^, the lifespan of the *rad2*C65Δ mutant decreased drastically whereas that of the WT and *rad2*C38Δ strains did not (Fig. 3D). Because of these observations pertaining to the UV-induced sterility and decreased lifespan of *rad2*C65Δ, it is possible that the pathogenesis of CS after DNA damage in patients with C-terminal truncations of XPG also involves actin cytoskeleton dynamics.

### Rad2p overexpression causes excessive cell polarity

**Fig. 4.**
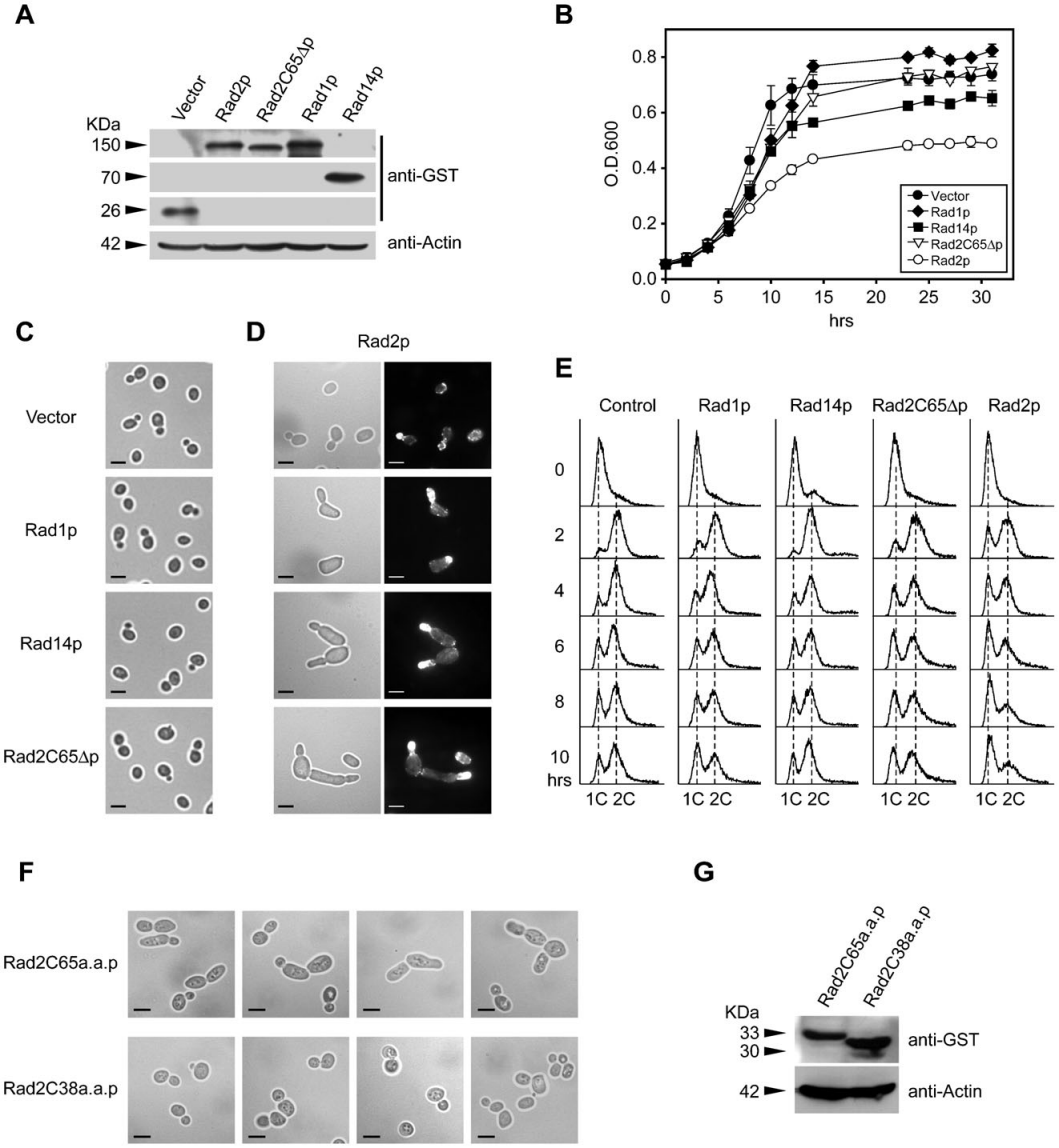
Effects of Rad2p and Rad2C65Δp overexpression. (A) Western blot analysis of Rad2C65p expression in *rad2*Δ cells, and Rad2p, Rad1p and Rad14p expression in WT cells. *RAD1*, *RAD2*, and *RAD14* were overexpressed in a WT background and *rad2*C65Δ was expressed in a *rad2*Δ background. The original autorads are presented in supplementary material Fig. S6. (B) Overexpression of the vector control, Rad1p, Rad14p and Rad2C65Δp did not cause acute growth defects. Rad2p overexpression significantly inhibited cell growth. Each curve represents an average of 3 or more experiments. (C) Overexpression of the vector control, Rad1p, Rad14p, and Rad2C65Δp did not cause any morphological changes. Bars, 5 µm. (D) Cell morphology was altered by Rad2p overexpression (left panel). Actin in Rad2p overexpressed cells is polarized at the region of growth, and long stretches of visible actin cable are visible in the elongated region of the cells (right panel). Bars, 5 µm. (E) Cell cycle progression was delayed by Rad2p overexpression, but was not affected by overexpression of the vector control, Rad1p, Rad14p, and Rad2C65Δp. The cells were synchronized by grown to stationary phase before cell cycle analysis. (F) Overexpression of the C-terminal 65 amino acids of Rad2 (Rad2C65a.a.p.) confers cell morphological alteration seen in panel D. Overexpression of the C-terminal 38 amino acids of Rad2 (Rad2C38a.a.p.) did not affect cell morphology. Bars, 5 µm. (G) Overexpression of Rad2C65a.a.p. and Rad2C38a.a.p. in the *rad2*Δ mutant background was verified by Western blot analysis.

The defective actin dynamics and cell polarization observed in *rad2*C65Δ mutant cells after UV irradiation suggest that Rad2p plays a role in cell polarity regulation. If so, Rad2p overexpression is expected to increase cell polarity. To test this hypothesis, the effects of *RAD1*, *RAD2*, *RAD14*, and *rad2*C65Δ expression were analyzed. Rad1p, Rad2p, and Rad14p were expressed in WT. However, Rad2C65Δp was expressed in *rad2*Δ mutant in order to avoid any possible phenotypic interference from the genomic Rad2p in WT background. We used *rad2*Δ mutant for all Rad2C65Δp overexpression related experiments through this study. However, we confirmed that overexpression of Rad2p or Rad2C65Δp in both WT and *rad2*Δ mutant brought about the same results in all the criteria we examined (supplementary material Fig. S5). This is probably due to low level of Rad2p from intact genomic *RAD2* during normal growth condition (846 molecules per cell) (Ghaemmaghami et al., 2003; Nicolet and Friedberg, 1987). The induced protein expression of these genes was first confirmed by Western blot analysis (Fig. 4A). Overexpression of the vector control, Rad1p, Rad14p and Rad2C65Δp did not cause acute growth defects or any morphological changes (Fig. 4B,C). In contrast, Rad2p overexpression induced early growth arrest (Fig. 4B) and distinct morphological changes, including single elongated cells and elongated budded cells (Fig. 4D, left panel). Actin in Rad2p overexpressed cells is polarized at the region of growth, and long stretches of actin cable are visible in the elongated region of the cells (Fig. 4D, right panel). Cell cycle progression was delayed in Rad2p-overexpressing cells, whereas cells overexpressing other genes showed a normal cell cycle pattern (Fig. 4E). To further verify the role of the 65 amino acids in the C-terminus of Rad2p, the *RAD2* sequences encoding either the last 65 or 38 amino acids were overexpressed (Fig. 4G). Expression of the C-terminal 65 amino acids of Rad2p alone caused distinct morphological changes that were similar to those of cells overexpressing normal Rad2p. However, these changes were not observed in cells overexpressing the C-terminal 38 amino acids of Rad2p (Fig. 4F). Taken together, these results demonstrate that the 65 amino acids in the C-terminus of Rad2p function in cell polarity and cell cycle regulation.

### Rad2p-induced hyper-polarized cell growth is suppressed by *tpm1*Δ

**Fig. 5.**
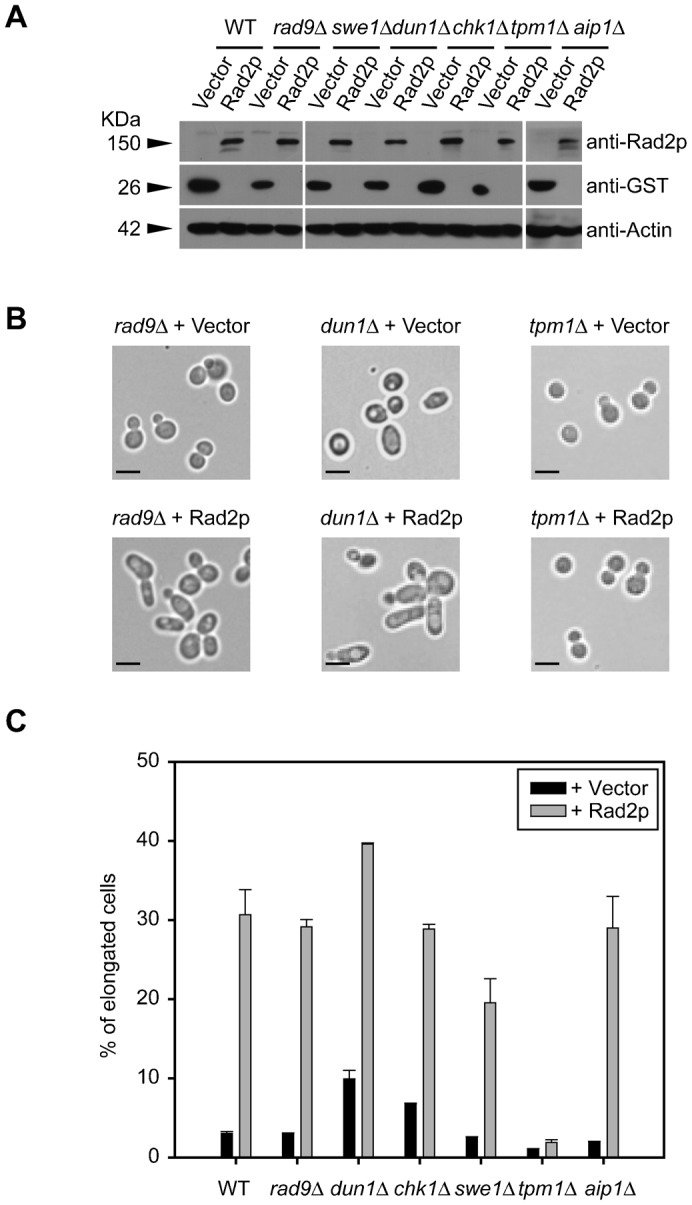
Effects of cell cycle-related genes on Rad2p-induced cell morphology alteration. (A) Overexpression of Rad2p in various deletion mutants. (B) The *rad9*Δ and *dun1*Δ mutations did not affect Rad2p-induced cell morphology. However, the *tpm1*Δ mutation completely abolished the hyper-polarized morphology of cells overexpressing Rad2p. Bars, 5 µm. (C) The percentage of cells elongated by overexpression of the vector control or Rad2p in mutants of various cell cycle and cell polarization-related genes. The number of elongated cells was counted among more than 1,000 cells of each strain and depicted as percentages. Each bar represents mean with standard deviations of 3 or more experiments. Solid bars and shaded bars indicate the vector controls and Rad2p overexpressing cells, respectively.

Given the effect of Rad2p function on cell polarization, we hypothesized that Rad2p overexpression would either enhance or suppress the phenotypes observed in cell polarization and cell cycle checkpoint mutants depending on the role of Rad2p in cell polarization. Rad2p was overexpressed in yeast harboring mutations in cell cycle checkpoint and cell polarization genes (Fig. 5A) and the percentage of elongated cells was assessed. The Rad2p-induced morphological change was not dependent on the 3 cell cycle checkpoint genes tested, *RAD9*, *DUN1* and *CHK1*. Conversely, the *swe1*Δ mutation led to a slight decrease in the Rad2p-induced elongated cell morphology. The most drastic morphological alteration was observed in the *tpm1*Δ mutant. Tpm1p is a tropomyosin that stabilizes actin and directs polarized cell growth through interactions with actin cables and filaments (Drees et al., 1995). The *tpm1*Δ mutation alone led to globular-shaped cells and a clear decrease in the number of elongated cells. Interestingly, the *tpm1*Δ mutation completely eliminated the elongated cell morphology of Rad2p overexpressing cells (Fig. 5B,C). The *aip1*Δ mutation is known to rescue the temperature sensitive growth and actin assembly defects of *tpm1*Δ mutant (Okada et al., 2006); however, it did not alter Rad2p overexpression-induced cell polarization (Fig. 5C), indicating that the function of *RAD2* in cell polarization is similar to that of *TPM1*, but it is not antagonized by *AIP1*. These observations support the critical role of *RAD2* in cell polarization.

### The *rad2*C65Δ mutation enhances the *tpm1*Δ phenotype

**Fig. 6.**
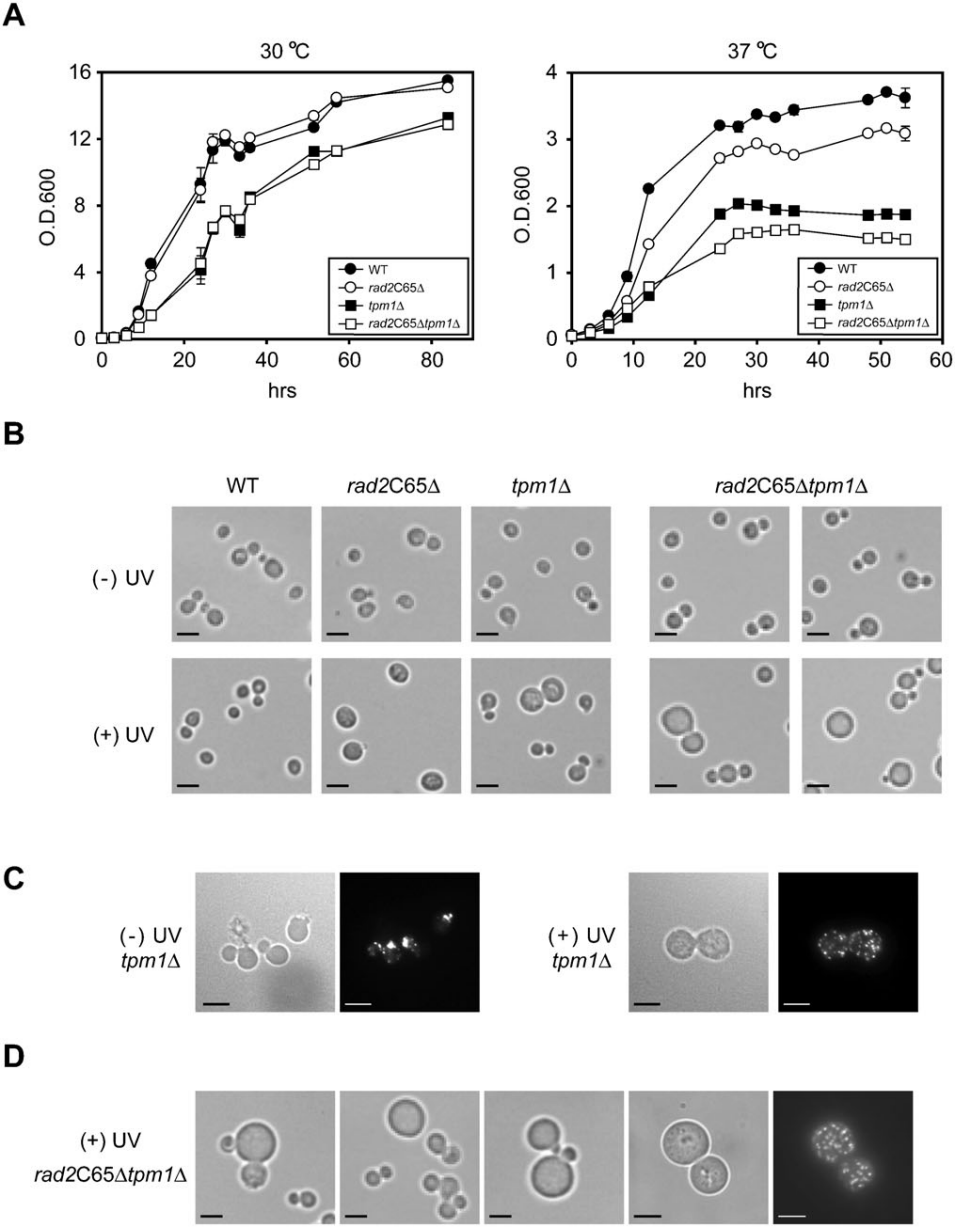
Phenotypes of the *rad2*C65Δ and *tpm1*Δ mutants. (A) Growth of the *rad2*C65Δ mutant at 30°C was similar to that of WT, whereas growth of the *tpm1*Δ mutant was slightly slower than that of WT. Growth of *rad2*C65Δ and *tpm1*Δ was slow at 37°C. The *rad2*C65Δ*tpm1*Δ double mutant had additively slowed cell growth at 37°C. Each curve represents the mean with standard deviations of 3 or more experiments. (B) The *rad2*C65Δ and *tpm1*Δ mutants were enlarged after 20 J/m^2^ of UV irradiation. The *tpm1*Δ mutant was more spherical and heterogeneous in cell size. Some cells of *rad2*C65Δ*tpm1*Δ mutant were larger than either of the single deletion mutants. Bars, 5 µm. (C) In the absence of UV, actin is polarized in *tpm1*Δ mutant. However, after UV irradiation, visible actin cables are decreased and actin patches are randomly distributed in enlarged *tpm1*Δ mutant cells. Bars, 5 µm. (D) Super-enlarged *rad2*C65Δ*tpm1*Δ mutant cells after UV irradiation have randomly distributed actin patches (right most panel). Bars, 5 µm.

The *tpm1*Δ mutant is characterized by slow growth at 30°C and temperature sensitivity at 37°C (Okada et al., 2006). Therefore, if *TPM1* genetically interacts with *RAD2*, the slow growth and temperature sensitivity of the *tpm1*Δ mutant would be aggravated by *rad2*C65Δ. Therefore, we examined the growth of single and double deletion mutants of *rad2*C65Δ and *tpm1*Δ. At 30°C, growth of the *rad2*C65Δ mutant was similar to that of WT, and the *rad2*C65Δ mutation did not exacerbate the slow growth of the *tpm1*Δ mutant (Fig. 6A, left panel). At 37°C, the growth of the *rad2*C65Δ and *tpm1*Δ single mutants was slow, and the growth of the *tpm1*Δ mutant was further delayed by the addition of a *rad2*C65Δ mutation (Fig. 6A, right panel). These results suggest that the function of Rad2p in cell polarization are insignificant under normal growth conditions but become important when growth conditions are stressed by environmental changes.

To further verify the function of Rad2p in actin polarization under DNA-damaging conditions, we examined the morphological changes in WT, *rad2*C65Δ, *tpm1*Δ, and *rad2*C65Δ*tpm1*Δ mutants before and after UV irradiation. In the absence of UV irradiation, the morphology of all 3 mutants was identical to that of WT (Fig. 6B). However, after UV irradiation, approximately 22% and 9% of the *rad2*C65Δ and *tpm1*Δ mutants, respectively, exhibited enlarged morphology. The *tpm1*Δ mutant was more spherical in shape and more heterogeneous in size than WT and *rad2*C65Δ cells. In addition, visible actin cables are decreased and actin patches are randomly distributed in enlarged *tpm1*Δ mutant cells (Fig. 6C) as in *rad2*C65Δ cells. In the *rad2*C65Δ*tpm1*Δ double mutant, the proportion of enlarged cells was additively increased to approximately 28% of the population (Fig. 6B), and some enlarged cells were significantly larger than those of the *rad2*C65Δ and *tpm1*Δ single mutants (Fig. 6D). After UV irradiation, as in the *rad2*C65Δ (Fig. 3B,C) and *tpm1*Δ (Fig. 6C) mutants, actin cables were undetected in the *rad2*C65Δ*tpm1*Δ mutant and actin patches were randomly distributed throughout the cell (Fig. 6D). These enhanced growth defects at 37°C and increased numbers of UV-induced enlarged cells of the *rad2*C65Δ*tpm1*Δ mutant indicate that *RAD2* has a function similar to that of *TPM1* in actin polarization.

### Rad2p promotes actin assembly

**Fig. 7.**
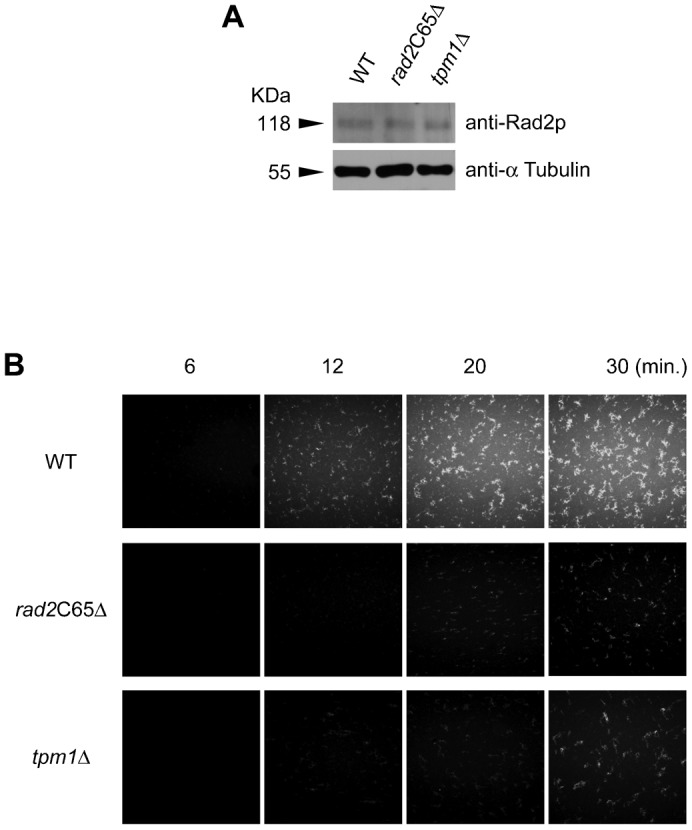
*In vitro* actin assembly assay. (A) Verification of Rad2p expression after UV irradiation. (B) The *rad2*C65Δ and *tpm1*Δ mutants exhibited considerably decreased actin assembly when compared to WT.

The *tpm1*Δ mutation decreases visible actin cables (Pruyne and Bretscher, 2000b); therefore, we tested the effect of Rad2p on *in vitro* actin polymerization rates using fluorescent tagged-actin monomers. After UV irradiation, Rad2p expression levels in *rad2*C65Δ and *tpm1*Δ cells were similar to that in WT cells (Fig. 7A). However, the actin polymerization rate of UV irradiated *rad2*C65Δ and *tpm1*Δ cell extracts was lower than that of WT extract (Fig. 7B). As expected from the results shown in Fig. 6B, the cell extract from the *tpm1*Δ mutant had only a slightly better actin assembly rate than that of the *rad2*C65Δ mutant (Fig. 7B). These results support a role for Rad2p in actin assembly.

## Discussion

### A role for the C-terminal 65 amino acids of Rad2p in the cell cycle and cell polarization

The drastically impaired growth of the *rad2*Δ and *rad2*CΔ mutants after UV irradiation suggests that the C-terminal region of Rad2p (last 38∼65 amino acids) is important for normal cell growth after UV exposure. The replicative lifespan of the *rad2*C65Δ mutant was drastically decreased after UV exposure, which is reminiscent of the crucial symptoms of XPG/CS patients such as growth retardation and premature aging that are aggravated after UV exposure. The cell cycle arrest observed in the *rad2*CΔ mutants after UV exposure suggests that the C-terminal region of Rad2p is essential for cell cycle regulation. In addition, a characteristic of the *rad2*C65Δ mutant observed after UV exposure was that an appreciable portion of the cell population exhibited an altered morphology with a single, rounded, and enlarged cell. Yeast mutants with polarization defects exhibit highly elongated or spherical buds due to excessive apical or isotropic growth, respectively. These abnormal morphologies are hallmarks of yeast growth polarization defects (Gourlay and Ayscough, 2005; Pruyne and Bretscher, 2000a; Pruyne and Bretscher, 2000b). The *rad2*C65Δ mutant also had a random distribution of actin patches accompanied by reduced visible actin cables and a complete loss of the normal response to α-factor after UV exposure. In contrast, overexpression of Rad2p induced hyper-polarization, a growth polarization defect with highly-elongated bud formation, whereas such a phenotype was not observed in Rad2C65Δp overexpressing cells. Furthermore, overexpression of the C-terminal 65 amino acids of Rad2p in the absence of DNA damage is sufficient to evoke actin cell polarization. These results further demonstrated the importance of the Rad2p C-terminal region in cell polarization. Conversely, overexpression of 2 other NER proteins, Rad1p and Rad14p, did not confer this hyper-polarization phenotype suggesting that the regulation of cell polarization is a unique function of Rad2p, and is independent of its function in NER.

Cell polarity is determined by the structure of the actin cytoskeleton in most eukaryotes, including yeast. Yeast actin is present in 2 types of F-actin structures, cortical patches and actin cables, where it appears as small dots and bundles of long actin filaments, respectively. Both actin structures are localized to the cell cortex and are implicated in the regulation of cell polarity and shape. During vegetative growth, these structures are polarized in a cell cycle-dependent manner. Maintaining actin cytoskeleton dynamics is crucial to cell survival, as mutations in genes involved in actin polarization decrease cell viability.

Genetic analysis using deletion mutants with cell cycle checkpoint or cell polarization defects revealed that hyper-polarization caused by Rad2p overexpression is independent of *RAD9*, *DUN1* and *CHK1* suggesting that Rad2p-induced cell polarization is not regulated by the *RAD9*-dependent cell cycle checkpoint pathway. In contrast, Rad2p-induced cell polarization is partially dependent on *SWE1* and is completely abolished by *tpm1*Δ. Swe1p functions at morphogenesis checkpoints by monitoring actin cytoskeleton perturbation (McMillan et al., 1998). Moreover, the lack of Rad2p-induced cell polarization in *tpm1*Δ mutant indicates that the actin stabilization function of Tpm1p is required for this polarization. In addition, the *rad2*C65Δ and *tpm1*Δ mutants exhibit decreased cell growth at 37°C, and augmented rounded enlarged cell morphology after UV exposure in a synergistic manner. Actin assembly was also decreased in both the *rad2*C65Δ and *tpm1*Δ mutants, suggesting that Rad2p is involved in actin structure regulation. Therefore, our results suggest that the C-terminal region of Rad2p has a unique function in morphogenesis checkpoints through cell polarization regulation. Because the effects of Rad2p on cell polarization are *RAD9*-independent, it is not clear whether Rad2p-induced cell polarization is related to or independent of damage-induced cell cycle regulation. However, based on the observation that Rad2p expression is increased in the presence of DNA damage (Robinson et al., 1986), the function of Rad2p in cell polarization regulation in response to DNA damage is likely a transient phenomenon. Taken together, we propose that the C-terminal region of Rad2p regulates the cell cycle and cell polarization.

### A link between XPG/CS and actin cytoskeleton dynamics

Besides its classical function in cell polarity, the actin cytoskeleton has been recognized as a crucial factor for many other cellular processes including cell cycle regulation, transcription, cell growth, survival, differentiation, skeletal muscle-specific gene expression, checkpoints and morphogenesis (Formigli et al., 2007; Hsu et al., 2010; Zheng et al., 2009). A recent study revealed that actin polymerization is important for proper cell cycle checkpoint control in human cells (Hsu et al., 2010). Moreover, abnormal cytoskeletal regulation is associated with human inherited neurodegenerative disorders (Goellner and Aberle, 2012; Nakamura et al., 2012). Given the fact that one of the prominent characteristics of CS is neurodegeneration, the defects in cytoskeleton dynamics observed in the yeast *rad2*C65Δ mutant is appealing way to explain the cause of CS.

Cell cycle arrest is commonly accompanied by cell polarity arrest, which is indispensable for morphogenesis and development in higher eukaryotes including humans. Cell polarity is determined by the structure of the actin cytoskeleton in most eukaryotes (Gourlay and Ayscough, 2005; Moseley and Goode, 2006; Pollard and Cooper, 2009; Pruyne and Bretscher, 2000a; Pruyne and Bretscher, 2000b). The reason why certain tissues such as neuronal cells and muscle cells are more influenced by CS has long been unknown. Recent studies have demonstrated that actin dynamics are critical for the induction of skeletal muscle-specific genes and the development of neuronal cell such as dendritic spines and Schwann cell myelination (Formigli et al., 2007; Hotulainen and Hoogenraad, 2010; Penzes and Rafalovich, 2012), which are preferentially deteriorated in CS patients. Indeed, a mouse CS model showed neurological symptoms due to defective myelination (Revet et al., 2012).

Nonetheless, the connection between actin dynamics and CS symptoms had not been previously investigated due to the lack of evidence indicating a role for XPG in the regulation of actin dynamics. Our observation of misregulated actin dynamics in the *rad2*C65Δ mutant after UV exposure provides evidence for this connection. In this study, morphological and cytoskeletal alterations of the *rad2*C65Δ mutant were observed only after UV irradiation, suggesting that XPG/Rad2p-associated cytoskeletal changes take place exclusively under DNA damage. Indeed, XPG expression is transiently increased by DNA damage in human cells (Tomicic et al., 2011). As the increased XPG expression is transient, the effect of XPG on actin dynamics is expected to be short-lived. Therefore, it might be difficult to observe the cellular effect of XPG on actin dynamics *in vivo*. However, in the case of an XPG C-terminal truncation mutation, XPG-mediated actin dynamics regulation would be delayed and actin regulation-related cellular mechanisms would consequently deteriorate.

### Rad2p C-terminal truncation as a cause of G1 cell cycle arrest

In addition to its well-known NER endonuclease activity, XPG recruits PCNA and is required for the DNA resynthesis step of NER (Staresincic et al., 2009). Rad2p also contains a potential PCNA-BD in the C-terminal region. The C-terminal truncation of Rad2p leads to failure of Rad2p-PCNA interaction, and the observation suggests a role of Rad2p PCNA-BD in cell cycle regulation (Kang et al., 2010). However, the PCNA-BD does not appear to be important for the cell cycle regulation function of CS-like phenotypes in yeast because *rad2*C38Δ did not confer a growth defect, lifespan decrease, or any morphological changes after UV damage. A study of a mouse XPG C-terminal truncation also showed that the last 183 amino acids (988–1170), including the PCNA-BD, did not induce XPG/CS phenotypes (Shiomi et al., 2004), indicating that the PCNA-BD itself is irrelevant to XPG/CS.

Contrary to the above-mentioned 183 amino acid deletion mutant, mice with a deletion of the last 360 amino acids (811–1170) of XPG exhibited XPG/CS phenotypes (Shiomi et al., 2004). The 183 amino acids deletion of the mouse XPG protein is similar to a 199 amino acids (988–1186) deletion of the human XPG protein (supplementary material Fig. S1B). This implies that the C-terminal region of the human XPG protein between amino acids 926 and 987 has cellular functions that are crucial for XPG/CS phenotypes because the minimal XPG C-terminal truncation found in an XPG/CS patient (XPCS1RO) is at amino acid 926. Recently, Werner syndrome protein (WRN) and XPG protein were shown to interact via their C-terminal regions. Although the direct interaction between XPG and WRN has been suggested to explain the severe developmental defect of XPG/CS patients, unidentified XPG functions were implied based on the greater severity of XPG/CS than Werner syndrome, a premature aging disorder onsets in adulthood. Indeed, WRN was shown to bind the very end of the XPG C-terminal region including the PCNA-BD (Trego et al., 2011), and the deletion of this region has been shown to not result in CS phenotypes in mouse (Shiomi et al., 2004). These observations indicate that the XPG C-terminal region upstream of PCNA-BD is responsible for XPG/CS. Likewise deletion of the region C-terminal to the PCNA-BD of Rad2p did not induce cell cycle arrest after UV exposure. This dispensability of these regions in XPG and Rad2p-PCNA-BD for CS-like phenotype indicates that an XPG region N-terminal to the PCNA-BD functions in cell cycle and cell polarization.

The defective association of XPG and TFIIH has attracted attention as a possible cause of CS. XPG is recruited to sites of DNA damage through the formation of stable complexes with TFIIH (Zotter et al., 2006). XPG C-terminal truncation mutations associated with XPG/CS are known to affect holo-TFIIH assembly, transcription, damage-induced RNA polymerase II degradation and transactivation of nuclear receptors. Therefore, the XPG C-terminal region is required for recruitment of XPG to core-TFIIH on DNA lesions and its deletion causes disassembly of holo-TFIIH by inhibiting CDK-activating kinase (CAK) recruitment (Arab et al., 2010; Ito et al., 2007). However, not all XPG C-terminal truncations in XPG/CS cells cause disassembly of TFIIH and CAK (Table 1). Instead, defective associations between XPG and TFIIH are uniform in XP/CS cells, indicating that defects in XPG-TFIIH interactions could be a radical cause of XPG/CS as mentioned above and/or CS caused by defects in other CS-related genes. If so, it is important to determine if the defective TFIIH-XPG interaction is the underlying cause of CS due to mutations in *CSA*, *CSB*, *XPB* and *XPD*. Core-TFIIH and CAK are well associated in CSA, CSB, and XPB/CS cells, and the failure of each mutant gene to interact with the core-TFIIH complex might not cause CS (Ito et al., 2007). However, CSB and XPB interact with the C-terminal region of XPG (Iyer et al., 1996; Sarker et al., 2005); therefore mutations in genes affecting XPG-CSB or XPG-XPB interactions might cause defects in TFIIH complex formation. In XPD/CS cells, XPG and the CAK complex are dissociated from TFIIH due to defects in the XPD protein (Ito et al., 2007). These results indicate that the dissociation of XPG from holo-TFIIH might be a radical cause of CS via a common factor that induces defects in cell cycle progression and actin cytoskeleton dynamics after DNA damage rather than a transcription defect and that XPG could be at the center of all known XP/CS.

**Table 1.**
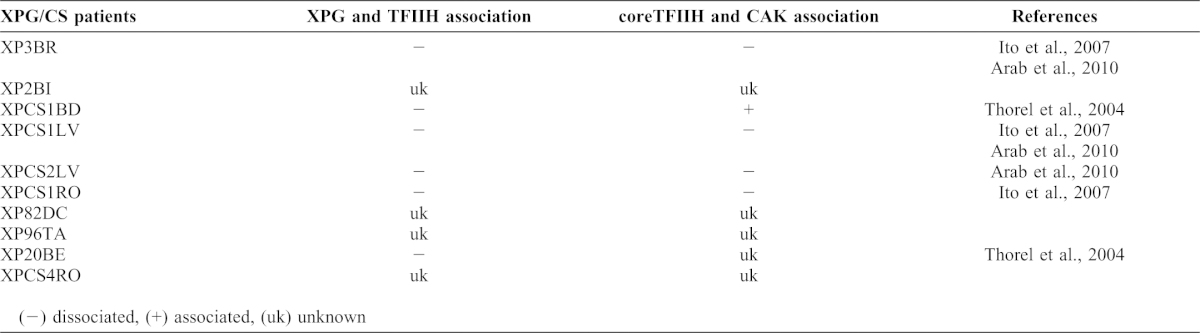
The association states of XPG-TFIIH and coreTFIIH-CAK in cells from XPG/CS patients.

Mutations in 2 TFIIH components, p44 and p52, also induce TFIIH destabilization but do not induce CS (Schärer, 2008a). This observation suggests that CS is not simply induced by TFIIH destabilization and that XPG dissociation is a possible cause of CS. Therefore, while the failure of XPG-CSB and XPG-XPB interactions likely cause defects in the stability of the holo-TFIIH complex, it is still possible that other mechanism(s) are involved in CSB and XPB/CS. A recent study revealed that XPD functions in cell cycle regulation via formation of CAK-XPD or *MMS*19-*MIP*18-*XPD* (MMXD) complex that does not contain any TFIIH components other than XPD (Ito et al., 2010). Therefore, the abrupt onset of CS and the exacerbation of CS symptoms after UV exposure (Lindenbaum et al., 2001) might be due to defects in cell cycle progression and actin cytoskeleton dynamics after DNA damage as observed in yeast. In other words, CS could be the result of a defect in cell cycle re-initiation after damage-induced cell cycle arrest and impaired regulation of actin dynamics.

### No increase in cancer incidence in CS

The UV sensitivity of XPG/CS patients might be the result of an NER defect. However, this UV sensitivity may occur in response to a defect in the recruitment of XPG to DNA lesions due to its failure to interact with TFIIH and/or form a stable repair complex rather than the endonuclease dysfunction of XPG (Arab et al., 2010; Ito et al., 2007; Zotter et al., 2006). Notwithstanding the UV sensitivity of XP and XP/CS, the characteristic phenotypes of these 2 disorders are very different. Although XP and XP/CS arise from mutations in the same genes, the characteristic symptoms are distinctly different. Most notably, sunlight-induced skin cancer incidence is 1,000 times higher in XP patients, whereas CS is not associated with an increased skin cancer incidence (Kraemer and DiGiovanna, 2003 (http://www.ncbi.nlm.nih.gov/books/NBK1397)). Elucidation of the functions of Rad2p in cell cycle regulation and actin polarization might help clarify the basis for the absence of increased cancer incidence in CS patients. The arrested cell division and decreased lifespan of *rad2*C65Δ mutant cells after UV exposure suggest that the C-terminally truncated XPG protein in XPG/CS cells causes cell cycle arrest and decreased cell longevity, and this resulted in the absence of increased cancer incidence in the presence of DNA damage in CS patients.

## Materials and Methods

### Yeast strains and plasmids

*Saccharomyces cerevisiae* WT strain EMY73 (*MATa his3-1 leu2-3*, *-112*, *ura3-52*, *trp1*Δ) and its isogenic derivative strains used in this study are described in supplementary material Table S1. To generate the *rad9*Δ, *chk1*Δ, *swe1*Δ, *dun1*Δ, *tpm1*Δ, and *aip1*Δ mutants, most open reading frames (ORF) were replaced with the *URA3* gene. Genomic deletion of these genes was confirmed by PCR using a pair of oligonucleotides flanking each ORF. For expression plasmid generation, ORFs of *RAD1*, *RAD2*, *RAD14*, *rad2*C65Δ, the *rad2*C65 amino acid coding region, and the *rad2*C38 amino acid coding region were cloned into pBJ842 to yield GST-*RAD1*, GST-*RAD2*, GST-*RAD14*, GST-*rad2*C65Δ, GST-Rad2C65a.a.p., and GST-Rad2C38a.a.p., respectively (Yu et al., 2011). The gene sequences and protein expression of each plasmid were confirmed by DNA sequencing and Western blot analysis, respectively.

### Determination of growth

Single yeast colonies grown on a synthetic complete medium lacking uracil were cultured overnight in the same fresh liquid medium. Cells were then diluted to OD_600_ = 0.05 in fresh medium, after which they were incubated at 30°C and the cell density was determined at the indicated time points using a spectrophotometer (DU650; Beckman Coulter, Inc., Brea, CA, USA). Protein overexpressing cells were then grown in leucine-depleted synthetic complete medium. For Fig. 6A, cells were grown in YPD medium (1% yeast extract, 2% peptone, and 2% yeast extract).

### UV sensitivity

UV sensitivity was analyzed as previously described (Kang et al., 2013).

### α-factor challenge

After overnight culture, cells were diluted in fresh YPD medium (pH 4.0) supplemented with 10 µg/mL α-factor and then incubated for 6 h at 30°C. For UV exposure, cells were UV irradiated immediately after dilution in fresh medium and incubated for 6 h at 30°C. For UV irradiation and α-factor treatment, cells were UV-irradiated, then treated with α-factor and incubated for 6 h at 30°C. Morphological changes were observed at room temperature using a fluorescence microscope (Zeiss Axioplan2; Zeiss, Germany) with 40× objective and 100× oil immersion objective lens. The images were captured using a SPOT FLEX digital capture system (Diagnostic Instruments, Sterling Heights, MI, USA).

### Actin cable and patch staining

The actin cytoskeleton was stained with rhodamine-conjugated phalloidin as previously described (Amberg, 1998) and then observed using a fluorescence microscope as described above.

### Cell cycle analysis

The cells were synchronized by grown to stationary phase. Then, the cells were diluted to OD_600_ = 0.05 in fresh medium. For DNA content analysis after UV damage, synchronized cells were diluted to OD_600_ = 0.05 in fresh medium and then UV irradiated. Aliquots were taken at the indicated time points and the cell cycle was analyzed by flow cytometry (FACSCalibur; BD Biosciences, San Jose, CA, USA) as previously described (Yu et al., 2011).

### Replicative lifespan analysis

The replicative lifespan was analyzed as previously described (Kennedy et al., 1994) using a micromanuplator (Singer MSM; Singer Instrument, UK). For samples subjected to UV irradiation, all procedures were the same except that the virgin mother cells were UV irradiated and incubated for 1.5 h in the dark before counting the buds. In each replicative lifespan assay, 60 cells were studied and daughter cells produced from a mother cell were counted.

### Western blot analysis

Whole cell extracts were prepared and Western blot analysis was performed as previously described (Yu et al., 2011). Expression of GST-tagged proteins was detected by using an anti-GST antibody (Calbiochem, Germany). Rad2p was detected using an anti-Rad2p antibody raised against a polypeptide corresponding to the N-terminus. Actin and α tubulin were detected as internal controls using anti-actin (Abcam, UK) and anti-α tubulin (Santa Cruz Biotechnology Inc., Santa Cruz, CA, USA) antibodies, respectively. The positive signal in the mock-transferred cells (vector) represents GST protein alone.

### *In vitro* actin assembly assay

*In vitro* actin assembly was evaluated as previously described (Soulard et al., 2002) except that the mixture was incubated at 37°C for 10 min, placed on a slide glass and periodically examined using a fluorescence microscope as described above.

## Supplementary Material

Supplementary Material
