# Supplementary material for: Yeast RAD2, a homolog of human XPG, plays a key role in the regulation of the cell cycle and actin dynamics
Source: Biol Open. 2013 Dec 4;3(1):29–41. doi: 10.1242/bio.20136403 (PMC3892158; doi:10.1242/bio.20136403)
Supplement: Supplementary Material [file supp_3_1_29__index.html]

Yeast RAD2, a homolog of human XPG, plays a key role in the regulation of the cell cycle and actin dynamics — Yeast RAD2, a homolog of human XPG, plays a key role in the regulation of the cell cycle and actin dynamics — Supplementary Material 

# Yeast *RAD2*, a homolog of human *XPG*, plays a key role in the regulation of the cell cycle and actin dynamics

## bio.20136403 Supplementary Material

**Files in this Data Supplement:**

- Supplementary Material - Mi-Sun Kang et al. doi: 10.1242/bio.20136403
