# Supplementary material for: Yeast RAD2, a homolog of human XPG, plays a key role in the regulation of the cell cycle and actin dynamics
Source: Biol Open. 2013 Dec 4;3(1):29–41. doi: 10.1242/bio.20136403 (PMC3892158; doi:10.1242/bio.20136403)
Supplement: Supplementary Material [file supp_bio.20136403_bio.20136403-s1.pdf]

Supplementary Material

Mi-Sun Kang et al. doi: 10.1242/bio.20136403

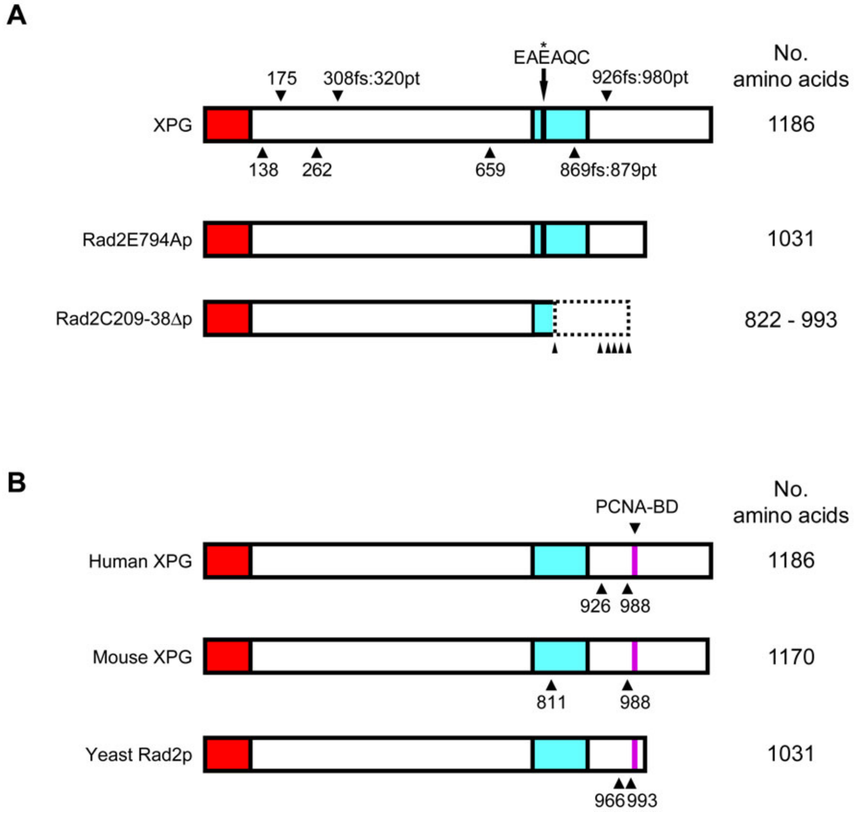

Fig. S1. Schematic representation of mutations in XPG and Rad2 proteins. (A) Arrowheads in XPG indicate the location of mutations in XPG where the protein terminates in cells isolated from 7 XPG/CS cases. The EAE*AQC sequence is identical in the XPG and Rad2 proteins and the E* residue in this sequence corresponds to the E791 residue in XPG and the E794 residue in Rad2p. In the *rad2CΔ* mutations, the positions of deleted amino acids in the Rad2 protein are marked with small arrowheads. fs: frame shift mutation, pt: protein termination, red box: N-terminal domain, greenish blue box: internal domain. (B) Mouse XPG protein is 16 amino acids shorter than human XPG. Purple box: PCNA-BD, arrow head: C-terminal truncation points in human and mouse XPG protein and in Rad2p described in discussion section.

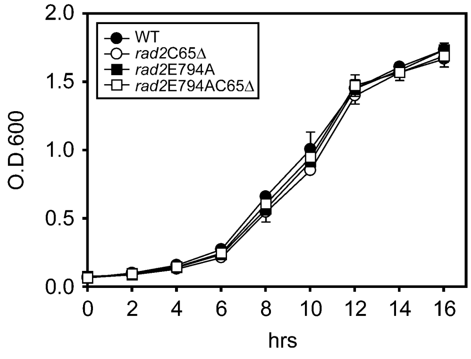

Fig. S2. Growth of WT and *rad2* mutant strains in the absence of UV exposure. Growth of all mutant strains was almost identical to WT.

Biology Open

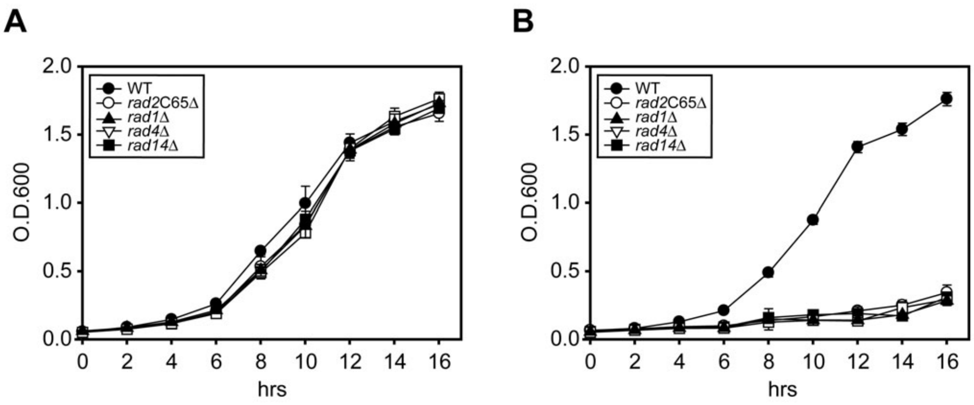

Fig. S3. Growth of WT and NER mutant strains. (A) Growth of all mutant strains was almost identical to WT in the absence of UV irradiation. (B) Growth of all mutant strains was drastically decreased compared to WT following 20 J/m^2 UV irradiation.

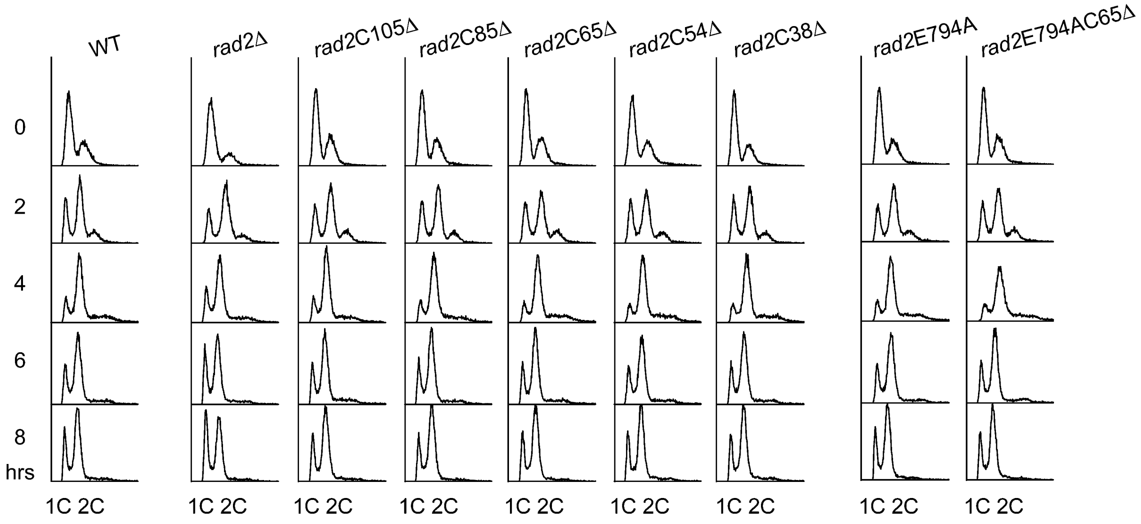

Fig. S4. DNA content analysis of WT and *rad2* mutants in the absence of UV exposure. After overnight culture, cell cycle progression was analyzed by flow cytometry. In the absence of UV irradiation, the cell cycle progression of all mutant strains was almost identical to that of WT.

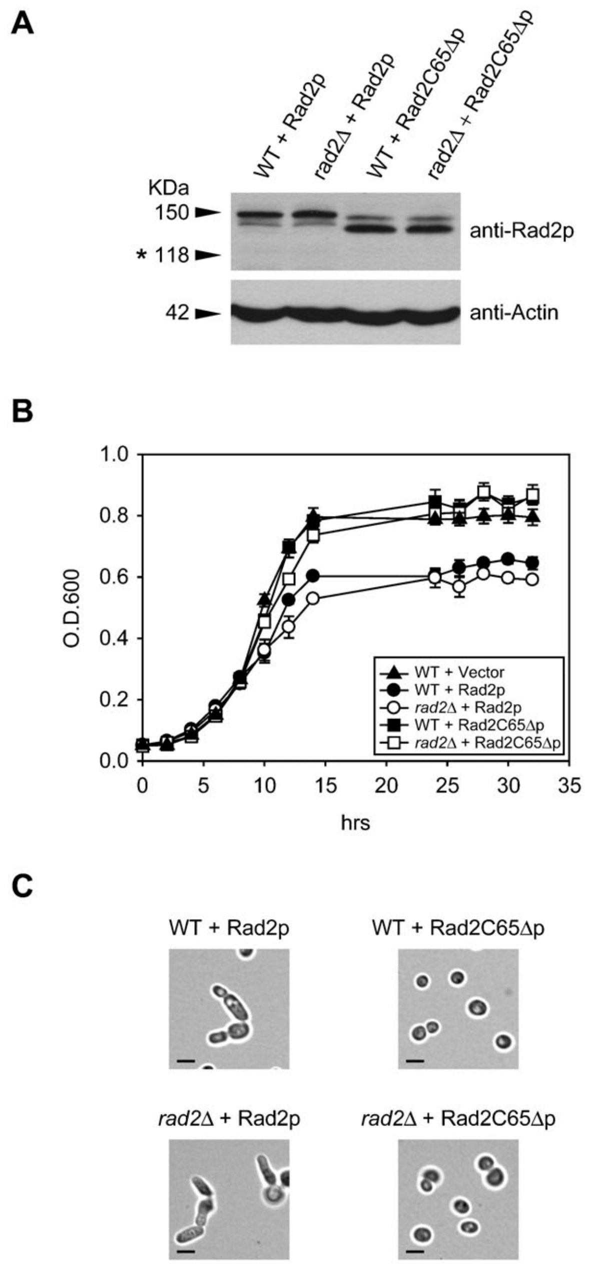

Fig. S5. Comparison of the effects of Rad2p and Rad2C65Δp overexpression in WT and the *rad2*Δ mutant. (A) Western blot analysis of Rad2p and Rad2C65Δp expression in WT and *rad2*Δ cells. Both Rad2p and Rad2C65Δp expression levels were similar in either WT or *rad2*Δ cells. Asterisk mark indicates the location of genomic DNA-originated Rad2p that was not detectable in cells grown under normal condition. (B) Overexpression of Rad2C65Δp did not evoke acute growth defects in both WT and *rad2*Δ cells. Rad2p overexpression significantly inhibited cell growth in WT and *rad2*Δ cells. Each curve represents an average of 3 or more experiments. (C) Cell morphology was altered by Rad2p overexpression in WT and *rad2*Δ cells. On the other hand, overexpression of Rad2C65Δp did not cause any morphological changes in WT and *rad2*Δ cells. Bars, 5 µm.

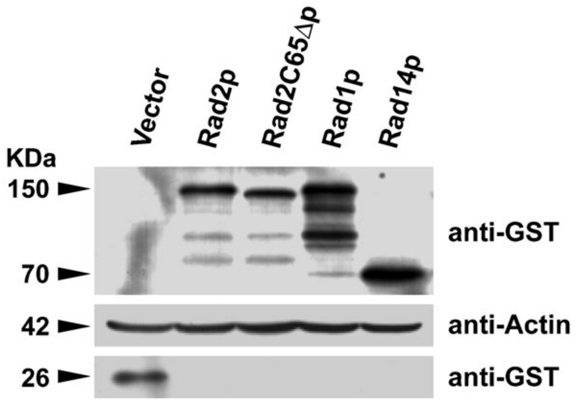

Fig. S6. The original autorads that were used for generating Fig. 4A.

Biology Open

Table S1. Yeast strains used in this study.

Strain	Genotype	Source
EMY73	MATa *his3*-Δ1*leu2*-3,-112 *ura3*-52, *trp1*::*URA3*	Lee et al., 2002a
EMY74.7	MATa *his3*-Δ1 *leu2*-3,-112, *ura3*-52, *trp1*Δ	Lee et al., 2002a
EMY75	EMY74.7 *rad2*Δ::*URA3*	Lee et al., 2002a
pLP9-1	EMY74.7 *rad2*C209Δ::*URA3*	Lee et al., 2002a
YSL207	EMY74.7 *rad2*C105Δ::*URA3*	This study
YSL255	EMY74.7 *rad2*C85Δ::*URA3*	This study
YSL254	EMY74.7 *rad2*C65Δ::*URA3*	This study
YSK1455	EMY74.7 *rad2*C54Δ::*URA3*	This study
YSL262	EMY74.7 *rad2*C38Δ::*URA3*	This study
YSK202-1	EMY74.7 *rad2*E794A::*URA3*	Lee et al., 2002a
YSK1410	EMY74.7 *rad2*E794AC65Δ::*URA3*	This study
YR1.62	EMY74.7 *rad1*Δ::*URA3*	Lee et al., 2002a
YR4.1	EMY74.7 *rad4*Δ::*URA3*	Lee et al., 2002b
YR14.42	EMY74.7 *rad14*Δ::*URA3*	Lee et al., 2002a
YSL873	EMY74.7 *tpm1*Δ::*URA3*	This study
YSL874	YSL254 *tpm1*Δ::*HIS3*	This study
YSL086	EMY74.7 transformed with pBJ842	This study
YSL087	EMY74.7 transformed with pR2.63	This study
YSL996	EMY74.7 transformed with pKR2.78	This study
YSL085	EMY74.7 *rad2*Δ transformed with pR2.63	This study
YSL258	EMY74.7 *rad2*Δ transformed with pKR2.78	This study
YKMS11	EMY74.7 transformed with pSKSL221	This study
YKMS27	EMY74.7 transformed with pSKSL607	This study
YSL511	EMY74.7 *rad2*Δ transformed with pSKSL313	This study
YSL515	EMY74.7 *rad2*Δ transformed with pSKSL315	This study
YSL834	EMY74.7 *rad9*Δ transformed with pBJ842	This study
YSL841	EMY74.7 *rad9*Δ transformed with pR2.63	This study
YSL835	EMY74.7 *swe1*Δ transformed with pBJ842	This study
YSL831	EMY74.7 *swe1*Δ transformed with pR2.63	This study
YSL836	EMY74.7 *dun1*Δ transformed with pBJ842	This study
YSL842	EMY74.7 *dun1*Δ transformed with pR2.63	This study
YSL837	EMY74.7 *chk1*Δ transformed with pBJ842	This study
YSL843	EMY74.7 *chk1*Δ transformed with pR2.63	This study
YSL838	EMY74.7 *tpm1*Δ transformed with pBJ842	This study
YSL832	EMY74.7 *tpm1*Δ transformed with pR2.63	This study
YSL839	EMY74.7 *aip1*Δ transformed with pBJ842	This study
YSL844	EMY74.7 *aip1*Δ transformed with pR2.63	This study
**Plasmids**		**Source**
pBJ842	GAL:GST 2μ *LEU2*d	Invitrogen
pR2.63	GAL:GST-*RAD2* 2μ *LEU2*d	Prakash
pKR2.78	GAL:GST-*rad2*C65Δ 2μ *LEU2*d	Prakash
pSKSL221	GAL:GST-*RAD1* 2μ *LEU2*d	This study
pSKSL607	GAL:GST-*RAD14* 2μ *LEU2*d	This study
pSKSL313	GAL:GST-*rad2*C65a.a. 2μ *LEU2*d	This study
pSKSL315	GAL:GST-*rad2*C38a.a. 2μ *LEU2*d	This study
